# 

**Published:** 1986-10

**Authors:** 


### BRISTOL MEDICO-CHIRURGICAL JOURNAL

VOLUME 101(v) OCTOBER 1986

NATIONAL LIBRARY
JUN 22 1987
OF MEDICINE

HYPERTENSION AND CORONARY ARTERY DISEASE

Appreciating Hypertension

Advice sheet for coronary patients

Do we tell our coronary patients enough?

The Bristol experience of coronary angioplasty

HEALTH CARE IN CHINA TODAY

IN ASSOCIATION WITH

BRISTOL MEDICINE

THE MEDICAL JOURNAL OF THE SOUTH WEST

